# The hidden risk of microplastic-associated pathogens in aquatic environments

**DOI:** 10.1016/j.eehl.2023.07.004

**Published:** 2023-07-21

**Authors:** Huan Zhong, Mengjie Wu, Christian Sonne, Su Shiung Lam, Raymond W.M. Kwong, Yuelu Jiang, Xiaoli Zhao, Xuemei Sun, Xuxiang Zhang, Chengjun Li, Yuanyuan Li, Guangbo Qu, Feng Jiang, Huahong Shi, Rong Ji, Hongqiang Ren

**Affiliations:** a School of Environment, Nanjing University, State Key Laboratory of Pollution Control and Resource Reuse, Nanjing 210023, China; b Department of Bioscience, Arctic Research Centre, Aarhus University, Roskilde, Denmark; c Higher Institution Centre of Excellence (HICoE), Institute of Tropical Aquaculture and Fisheries (AKUATROP), Universiti Malaysia Terengganu, 21030 Kuala Nerus, Terengganu, Malaysia; d Department of Biology, York University, 4700 Keele Street, Toronto, ON M3J 1P3, Canada; e Tsinghua Shenzhen International Graduate School, Shenzhen 518055, China; f State Key Laboratory of Environmental Criteria and Risk Assessment, Chinese Research Academy of Environmental Sciences, Beijing 100012, China; g Yellow Sea Fisheries Research Institute, Chinese Academy of Fishery Sciences, Qingdao 266071, China; h Institute of Environmental Research at Greater Bay Area, Key Laboratory for Water Quality and Conservation of the Pearl River Delta, Ministry of Education, Guangzhou University, Guangzhou 510006, China; i Key Laboratory of Environment and Health (HUST), Ministry of Education & Ministry of Environmental Protection, and State Key Laboratory of Environmental Health (Incubation), School of Public Health, Tongji Medical College, Huazhong University of Science and Technology, Wuhan 430030, China; j State Key Laboratory of Environmental Chemistry and Ecotoxicology, Research Center for Eco-Environmental Sciences, Chinese Academy of Sciences, Beijing 100085, China; k School of Environmental Science and Engineering, Guangdong Provincial Key Lab of Environmental Pollution Control and Remediation Technology, Sun Yat-sen University, Guangzhou 510275, China; l State Key Laboratory of Estuarine and Coastal Research, East China Normal University, Shanghai 200241, China; m University Centre for Research and Development, Department of Chemistry, Chandigarh University, Gharuan, Mohali, Punjab, India

**Keywords:** Microplastics, Pathogens, Colonization, Transmission, Public health

## Abstract

Increasing studies of plastisphere have raised public concern about microplastics (MPs) as vectors for pathogens, especially in aquatic environments. However, the extent to which pathogens affect human health through MPs remains unclear, as controversies persist regarding the distinct pathogen colonization on MPs as well as the transmission routes and infection probability of MP-associated pathogens from water to humans. In this review, we critically discuss whether and how pathogens approach humans via MPs, shedding light on the potential health risks involved. Drawing on cutting-edge multidisciplinary research, we show that some MPs may facilitate the growth and long-range transmission of specific pathogens in aquatic environments, ultimately increasing the risk of infection in humans. We identify MP- and pathogen-rich settings, such as wastewater treatment plants, aquaculture farms, and swimming pools, as possible sites for human exposure to MP-associated pathogens. This review emphasizes the need for further research and targeted interventions to better understand and mitigate the potential health risks associated with MP-mediated pathogen transmission.

## Highlights

• Pathogenic bacteria and viruses colonized on microplastic (MP) surfaces are reviewed.
• Polymer types of MPs affect the major pathogen species colonized on MP surfaces.
• Human ingestion of MPs leads to the ingestion of pathogens coated on MP surfaces.
• Lack of data hinders quantifying potential health risks from MP-associated pathogens.

## Graphical abstract

**Figure:**
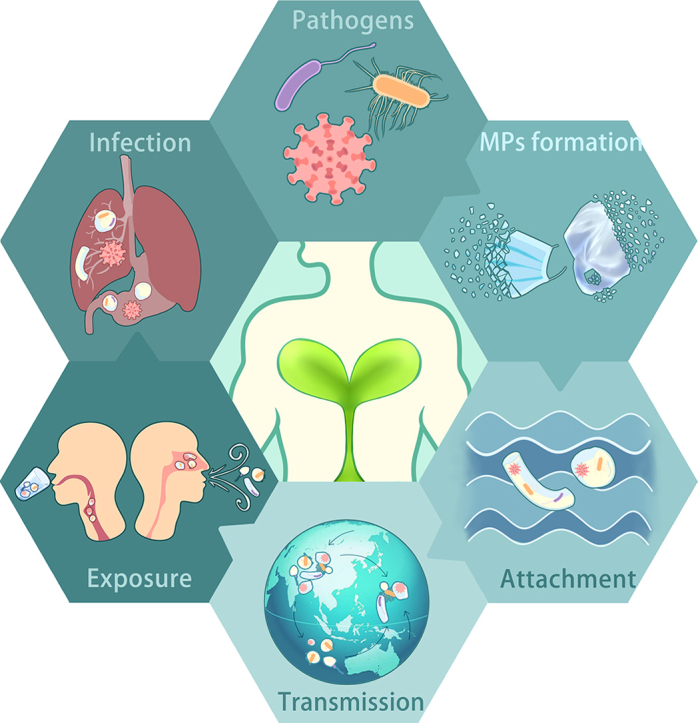

## 1 Introduction

The production of plastics has exceeded most other artificial materials manufactured since the 1950s, and it is estimated that 11% of plastic waste produced in 2016, i.e., approximately 19–23 million tons, entered aquatic environments [1,2]. Large plastics undergo weathering in the environment and slowly break down into small particles known as secondary microplastics (MPs) if less than 5 mm [3]. Some plastic items are also intentionally produced as primary MPs, such as nurdles, industrial raw material for plastic product manufacturing [4].

Tiny MPs are readily consumed by humans with annual ingestion of 39,000–52,000 MPs/individual in America, according to an estimate based on caloric and water intake [5]. This draws growing attention to the deleterious effects of this emerging contaminant on human health. Increasing research suggests possible physical, chemical, and biological toxicity induced by both MPs and the adsorbed toxins, including potential pathogens, but comprehensive assessments of the toxicity at environmentally relevant concentrations and for different types of plastics are still lacking [[6], [7], [8]]. Pathogens in aquatic environments can originate from anthropogenic sources, such as the effluents discharged from wastewater treatment plants (WWTPs) and the release of contaminated sanitary sewer flows, and natural sources such as the excreta from livestock and wildlife [9]. Although the health concerns raised by the so-called Trojan horse effect of MPs remain controversial, an array of studies demonstrates that pathogenic bacteria, such as *Vibrio*, *Escherichia coli* (*E. coli*), and *Acinetobacter baumanii* colonize MPs in water bodies [10,11]. Moreover, viruses, such as poliovirus, norovirus, and coronavirus, are found to persist on plastic surfaces for several days [[12], [13], [14]]. Nevertheless, research on the risk of pathogen-attached MPs to humans is still in its infancy and requires further investigation.

In assessing the potential detrimental impacts of pathogen-attached MPs from the aquatic environment on human health, three critical and interrelated questions should be addressed: (i) Do pathogens preferentially adhere to MPs in aquatic environments? (ii) How are MP-associated pathogens transmitted from the aquatic environment to humans? (iii) Can exposure to MP-associated pathogens lead to human infection? Although timely and important, some previous reviews have focused more on the impact of MP-associated pathogens on wildlife health rather than on humans or only discussed the indirect effects on human health via the food chain and antimicrobial resistance genes [[15], [16], [17], [18], [19]]. Some other reviews have discussed the health risk of MP-associated pathogens by extrapolating from the evidence on macroplastics without analyzing the entire process by which MP-associated pathogens come into contact with humans and cause potential infection [[20], [21], [22]]. Importantly, about 80% of the research on pathogen transmission via MPs has been published since 2021, partly due to the COVID-19 pandemic, which has led to the widespread use of disposable masks, gloves, and other personal protective equipment, as well as increased public concern about both MPs and pathogen transmission [23]. This makes it difficult for previous reviews to comprehensively analyze the health risks of MP-associated pathogens to humans.

To address these gaps and provide a more comprehensive assessment of the health effects of MP-associated pathogens on humans, this review aims to discuss the abovementioned three questions. By leveraging recent advancements in understanding human exposure to MP-associated pathogens, we seek to elucidate the interactions among MPs, potential pathogens, and humans. This knowledge will facilitate informed mitigation decisions to combat contagious diseases potentially caused by MP-associated pathogens and enable accurate risk predictions associated with MPs.

## 2 Search strategy and selection criteria

**Fig. 1:**
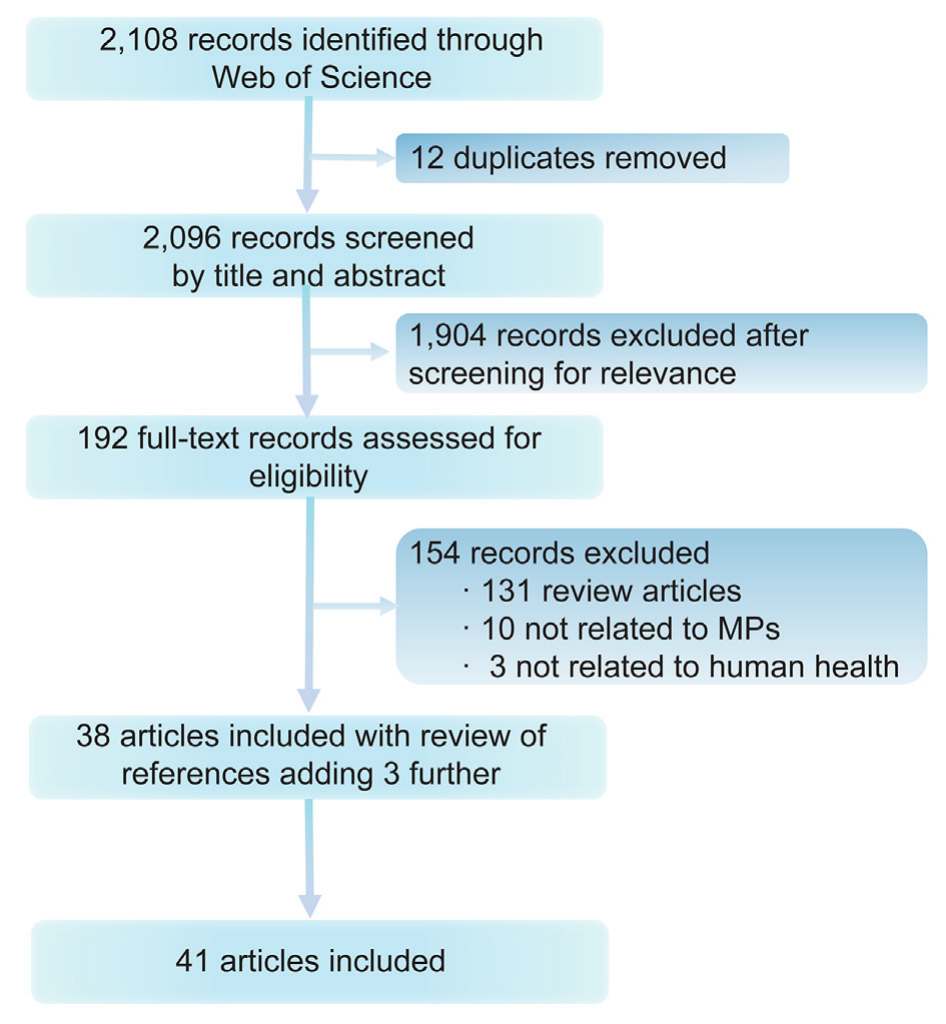
A flowchart describing the study selection process. MPs, microplastics.

Web of Science Core Collection was searched on August 22, 2022, for articles published in English, and there was no restriction on publication years. The searching formula “TS = (plastic∗) AND (pathogen∗ OR bacteria∗ OR fung∗ OR virus∗) AND (coloni∗ OR attach∗ OR adsorb∗ OR lade∗ OR associate∗ OR vector∗ OR hitchhiker∗ OR trojan horse∗) AND (transmit∗ OR deliver∗ OR spread∗ OR disseminate∗ OR propagat∗) AND (disease∗ OR human health)” were used, and a total of 2,108 papers were yielded, of which 12 duplicates were removed. We scanned the title and abstract of these 2,096 records to exclude irrelevant articles (n = 1,904). We identified irrelevant articles as studies that investigated macroplastics or plastic debris with no size range, studies that were not related to plastics at all (e.g., using plastic containers or materials to perform experiments), and publications that did not report any primary data or did not present new analyses of existing data. We then read the full text of the rest 192 articles, further removing 154 records. The remaining 26 articles together with 7 more articles screened from their reference lists were included in this paper (Fig. 1).

## 3 Attachment of pathogens to MPs in aquatic environments

### 3.1 Pathogen attachment

In the aquatic environment, the attachment of pathogens to MPs is a complex process. First, changes in surface morphology and chemistry immediately occur when MPs enter the aquatic environment, which can facilitate the adsorption of substances, such as organic matter and nutrients, from the surrounding water [24]. These altered surfaces provide an attractive environment for microorganisms, leading to the formation of a plastisphere [11]. The identification of associated bacteria and viruses is often studied using metagenomic sequencing of deoxyribonucleic acid (DNA) or ribonucleic acid (RNA). Additionally, the persistent nature of MPs in different aquatic environments serves as a long-term substrate for microbial growth. In the marine environment, for instance, the estimated half-life of high-density polyethylene ranges from 58 to 1,200 years [25]. In contrast, microbial attachment to plastic surfaces occurs within minutes, with a stable plastisphere formed within 6 weeks [26]. Some polymer types, such as polyethylene terephthalate, can be degraded by specific microorganisms, enhancing the survival and growth of certain microbes [[27], [28], [29], [30]]. Meanwhile, readily biodegradable polymers (i.e., polymers that are broken down by bacterial degradation) and plastic additives (e.g., plasticizers and flame retardants) commonly added to plastics can also provide additional carbon sources to stimulate the enzymatic activity of certain microorganisms [[31], [32], [33], [34]].

Biofilm formation on the surface of MPs is another crucial aspect of pathogen colonization. When pristine MPs enter the aquatic environment, an adsorbed layer of biomolecules and natural organic matter, known as the eco-corona, rapidly forms on their surfaces [24]. This eco-corona provides primary nutrients for microbial growth and serves as a foundation for biofilm development [35]. Biofilms on the surface of MPs act as a shield, enabling microorganisms, including pathogens, to withstand adverse environmental conditions and disperse to new niches [36]. Microorganisms living within biofilms, including pathogens, employ a combination of physiological adaptations, physical protection, resource sharing, genetic exchange, communication, and microenvironmental heterogeneity to better cope with environmental stressors, such as antibiotics and ultraviolet light [[36], [37], [38], [39]]. These strategies allow them to adapt to changing environments and increase their chances of survival compared to free-living microorganisms. Biofilms also facilitate the accumulation, development, and spread of antibiotic resistance genes (ARGs), allowing the survival of harmful microbes under antibiotic treatment [40]. Moreover, extracellular polymeric substances (EPS) secreted by microorganisms are the fundamental constituents of biofilms and play a crucial part in attracting other microbes to MPs [41]. The EPS immobilize microbial cells, allowing communication, cooperation, and competition among microorganisms [42]. Both EPS and nutrients from the surrounding environments serve as energy sources for microorganisms [42,43].

**Fig. 2:**
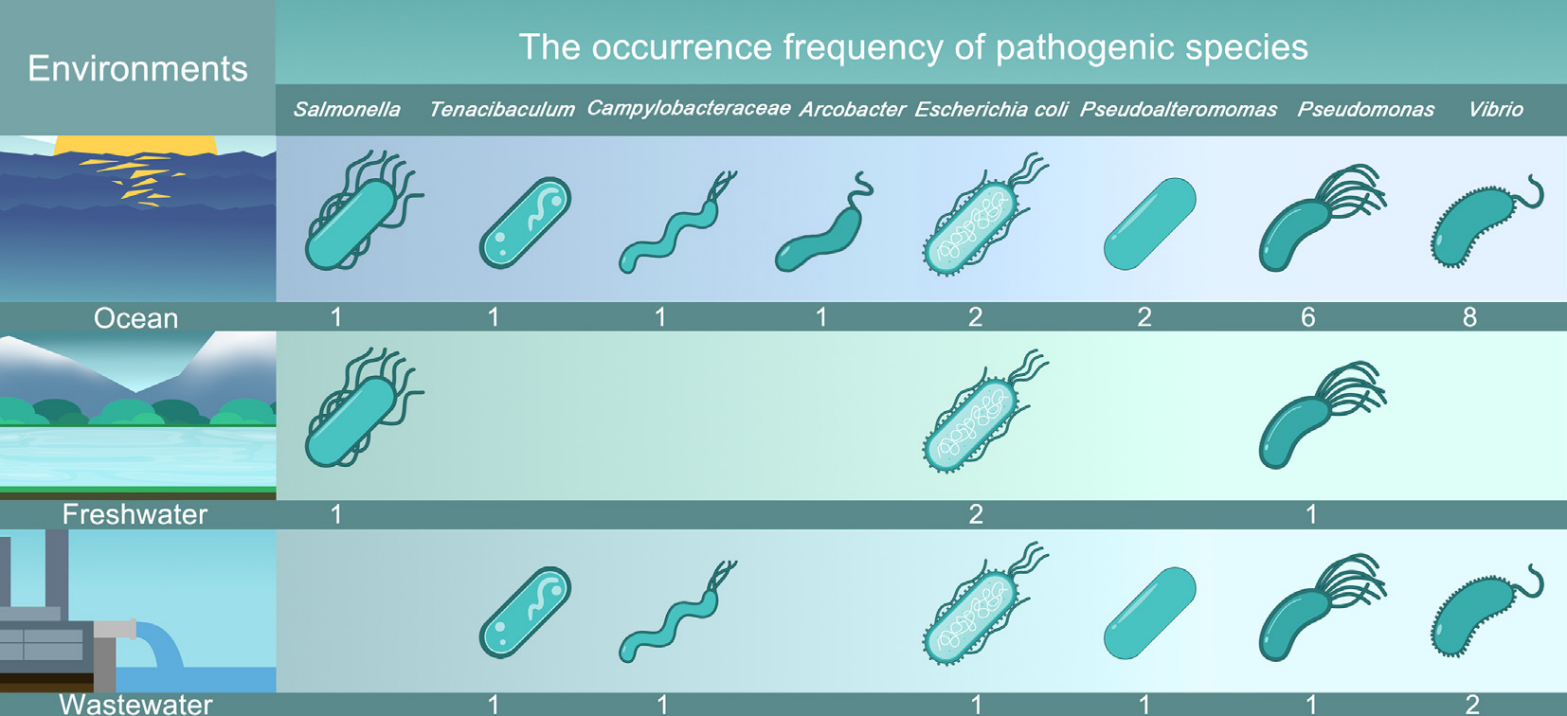
Eight common and potentially pathogenic bacteria detected on MPs in different aquatic ecosystems based on literature search results [[46], [47], [48], [49], [50], [51], [52], [53], [54], [55], [56], [57], [58], [59], [60]]. Numbers below the pathogenic bacteria indicate their frequency of occurrence in all retrieved studies.

MP-associated pathogens in aquatic environments are highly diverse, posing a potential risk of transmission from animal guts or WWTPs to other ecosystems. Fig. 2 provides an overview of eight common pathogenic bacterial species on MPs in different aquatic ecosystems. These pathogenic bacteria are classified into three groups based on the environments in which they were detected: ocean, freshwater, and wastewater environments. Previous studies have primarily focused on the colonization of potentially pathogenic bacteria on MPs in the marine environment, with Vibrio bacteria being the most frequently detected species in different aquatic environments. It is worth noting that the abundance of pathogens varies over spatiotemporal scales. For example, the prevalence of the genus *Vibrio* on MPs in the summertime and the bacterial species richness on MP surfaces decrease with the increasing latitudes in the Northern Hemisphere [44,45].

In addition to bacteria, viruses can also survive on plastic surfaces, and several studies have investigated the viral load and duration of persistence on plastic surfaces (Table 1). The survival time alters depending on surface properties, ranging from hours to days. For example, flu viruses (influenza A and B) remained infectious much longer on steel and plastic surfaces (24–48 h) than on cloth, paper, and tissue surfaces (less than 8–12 h) [61]. Similarly, a recent study suggested that the severe acute respiratory syndrome coronavirus 2 (SARS-CoV-2) was more stable on plastics and stainless steel than on copper and cardboard, with an inactivation time of about 72 h [62]. Moreover, the SARS-CoV-2 RNA persisted on KN95 facial masks and medical isolation gowns for 5–30 days and remained infectious for about 5–7 days [63]. The presence of various pathogens on MPs indicates a potential for cross-ecosystem transmission, causing hidden risks for human health that require further investigation, as discussed in the subsequent sections.

**Table 1:** Summary of viruses on plastic surfaces.

Virus type	Strain	Final viral titer TCID_50_/mL	Temperature/°C	Relative humidity/%	Persistence/h	Reference
SARS-CoV-1	HKU39849	10^6^	22–25	40–50	> 72	[64]
	CoV–P9	NA	20	NA	∼72	[65]
	Isolate FFM1	<10^2^	21–25	NA	∼216	[66]
	AY274119.3	10^0.7^	21–23	40	∼72	[62]
SARS-CoV-2	NA	2.27 ± 0.09	22	65	96	[67]
	MN985325.1	<1	23	40	72	[62]
Influenza virus	A/Brazil/11/78-like, H1N1	1	26.7–28.9	55–56	48	[61]
	B/Illinois/1/79-like					
	Adapted A/Puerto Rico/8/34	10^2^	17–21	23–24	∼4	[68]
	Isolate A/Cambridge/AH04/2009					
MERS-CoV	Isolate HCoV-EMC/2012	<1	20	20–40	48	[69]
Human coronavirus	Human coronavirus 229E	<1	21	30–40	120	[70]

SARS-CoV-1, severe acute respiratory syndrome coronavirus1; SARS-CoV-2, severe acute respiratory syndrome coronavirus2; MERS-CoV, Middle East respiratory syndrome coronavirus; TCID_50_, tissue-culture infectious dose; NA, not available.

### 3.2 Factors affecting pathogen attachment to MPs in the aquatic environment

**Fig. 3:**
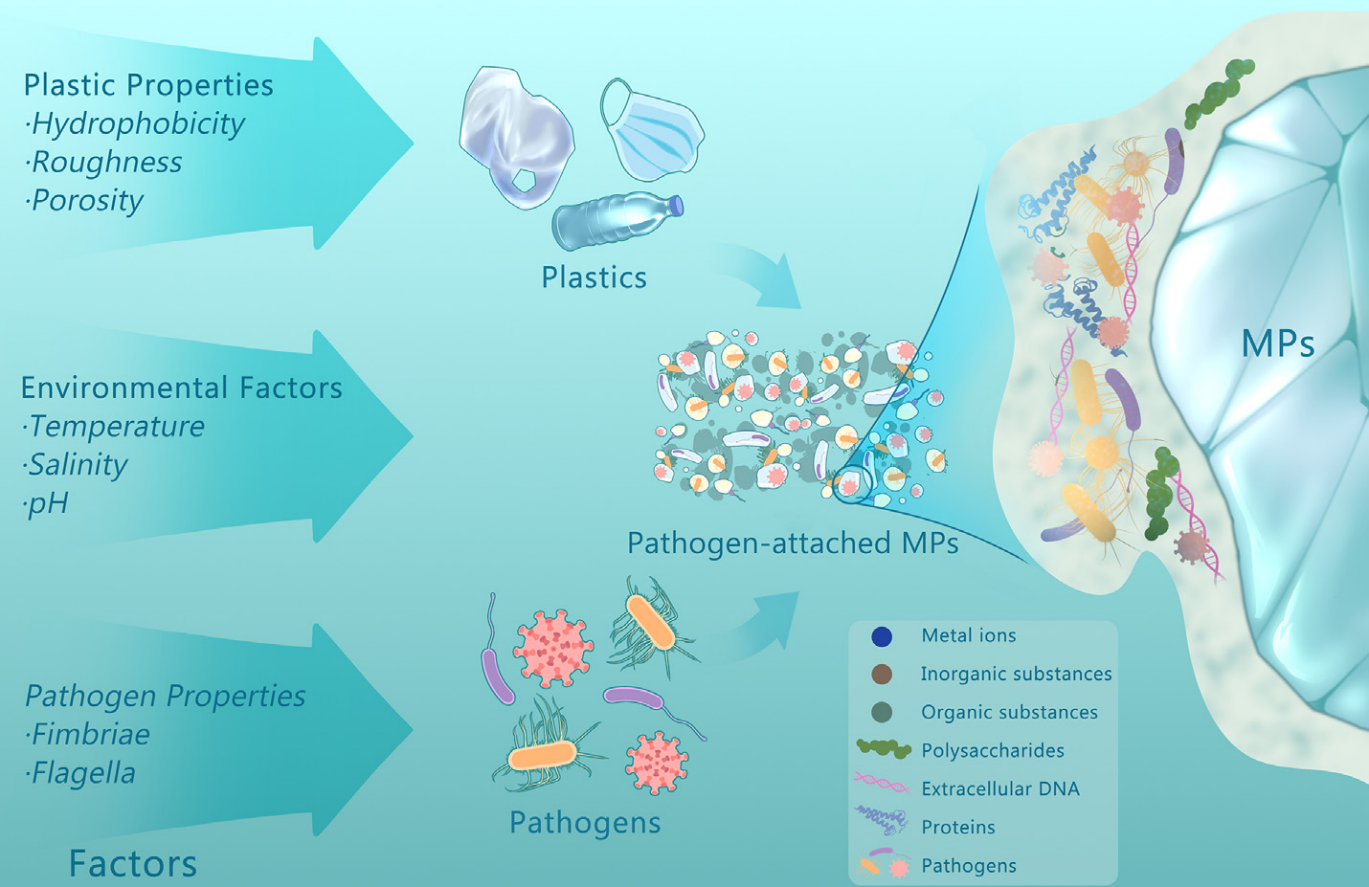
Factors affecting the early microbial colonization on MPs in aquatic environments.

The attachment of pathogens to MPs is influenced by several factors, including the properties of both the MPs and the pathogens, as well as environmental factors. These factors determine the subsequent colonization process of pathogens on MPs (Fig. 3). The hydrophobicity, roughness, and porosity of MPs are relevant to pathogen attachment [71]. Hydrophobic MPs provide a solid interface for the adsorption of substances such as organic matter, which subsequently attracts microbes for colonization. This hydrophobic interaction between microorganisms and MPs allows microorganisms to overcome repulsive forces between the microbial cells and the surfaces of MPs to avoid detachment [20,71]. Environmental weathering of plastic debris can increase their surface sites for pathogen colonization and enhance nutrient adsorption. Besides, porous MPs prolong the persistence of pathogens, including viruses, compared to non-porous surfaces [37,72].

Bacterial adhesion is another crucial factor benefiting the attachment to MPs in aquatic systems. Most pathogenic bacteria possess fimbriae (short pili extending from the cytoplasmic membrane of bacteria) that can facilitate their adhesion to environmental surfaces [73]. The fimbriae promote the attachment of pathogenic bacteria to MPs by overcoming the initial electrostatic repulsion between bacteria and MPs [74]. Bacterial flagella can also excrete adhesive proteins to help them attach to surfaces and overcome repulsive forces associated with the substratum [71]. However, it should be noted that these MP properties also favor the attachment of nonpathogenic organisms, and the specific effects of non-pathogenic microorganisms on pathogen attachment to MPs vary depending on the composition of microbial communities, environmental conditions, and the characteristics of the pathogenic and non-pathogenic species involved.

In addition to the properties of MPs and pathogens, environmental factors, such as temperature, salinity, and pH, are also critical to the survival and proliferation of pathogens on MPs in aquatic environments. These factors can have varying effects depending on the specific pathogen species involved [75]. Low temperatures (e.g., 4–6 °C) favor prolonged persistence of pathogens, such as *E. coli* and *Salmonella typhimurium (S. typhi)*, on abiotic surfaces, including MPs [11]. Decreases in salinity have also been observed to increase pathogen survival, as higher salinity can induce an osmotic upshock that inhibits several transmembrane transport systems in pathogens [76]. The near-neutral pH of 6.5–7.0 is suitable for the growth of most bacteria, including pathogenic types, yet pathogenic enteric bacteria, including *E. coli*, *Helicobacter pylori*, and *S. typhi*, can survive the low pH of the stomach [77]. Nevertheless, the effect of pH on viral survival is a different story because viruses remain stable over a wide range of pH values, and the role of pH in viral persistence on surfaces is associated with the alteration in viral surface charge [78].

### 3.3 Controversy on preferred attachment to MPs

There is ongoing debate as to whether microbes preferentially colonize MPs, although it is widely recognized that diverse microbes can attach to MPs [11]. The disagreement is partly due to a lack of holistic understanding of the factors affecting microbial attachment to MPs in water, as discussed above, as well as insufficient data in this regard. In general, the proclivity of microorganisms to attach to a surface depends on the selective advantage that the surface provides, including hydrophobicity, roughness, surface charge, and degradability [36]. These features possibly explain the preferential colonization of specific microbes on MPs. Several studies have suggested that certain microbes, including pathogenic species, are more abundant on MPs than on non-plastic particles. For example, in different aquatic environments, such as seawater and freshwater, the abundance of the Hyphomonadaceae and Erythrobacteraceae families is significantly higher on polystyrene and polyethylene than that on wood pellets [79]. In addition, the abundance of *Burkholderiales*, some of which are pathogenic, is more than twice as high on MPs than other substrates [80]. Furthermore, the specific accumulation of ARGs by MPs is evidenced by the fact that MPs have higher average absolute abundances of most ARGs than the surrounding sediment samples [40].

On the other hand, it is also speculated that the observed differences between the attachment of microbes on MPs and that on natural particles (e.g., wood, rock, and leaf) could be attributed to the non-selective colonization of microorganisms [21]. MPs and natural particles show notable differences in α-diversity and β-diversity in different studies. These differences, however, likely result from the different sample data sets instead of the actual sample characteristics in each study [18]. Some other studies investigating the α-diversity of MPs show that there is no difference between the microbial diversity of MPs and the surrounding aquatic environments [81,82]. Oberbeckmann and Labrenz [18] employed a β-diversity analysis and demonstrated that the MPs-colonizing bacteria did not distinctively differ from those colonizing natural surfaces, such as wood, cellulose, and glass. Furthermore, the specificity of biofilm and core microorganisms on MPs remains uncharacterized, making it difficult to compare the microbial communities between MPs and natural particles [83].

To sum up, the controversy remains over whether microorganisms prefer colonizing MPs to natural particles in aquatic environments, as the above comparison of both sides is based upon limited data. Therefore, to end the controversy, it is urgent to quantitatively compare the abundance of microbes, including pathogens, on MPs and the surrounding natural substrates, and this quantitative comparison must rely on sufficient field data.

## 4 Transmission of MP-associated pathogens in aquatic environments

### 4.1 Transmission possibility

Transmission of pathogens via MPs can occur in a theoretical manner. Research has provided evidence that marine plastic debris has the potential to introduce non-native microbial species to a new environment [84]. Moreover, microorganisms are less affected by the alteration in geography, and their communities are not limited by dispersal [85,86]. The interaction between MPs and microorganisms may allow the alteration in microbial communities and the occurrence of horizontal gene transfer and pathogen transmission. It has been demonstrated that changes in microbial communities on MPs in aquatic environments across different locations and the detection of pathogenic microorganisms in areas where they are not typically found [20,87,88]. Moreover, microbial communities colonized on MPs can be sensitive to temporal variations due to the changes in temperature, which may lead to different microbial composition results [44,45]. Notably, transport and dilution of MPs impact pathogen abundance, with dilution in large water bodies decreasing MP-associated pathogens.

**Fig. 4:**
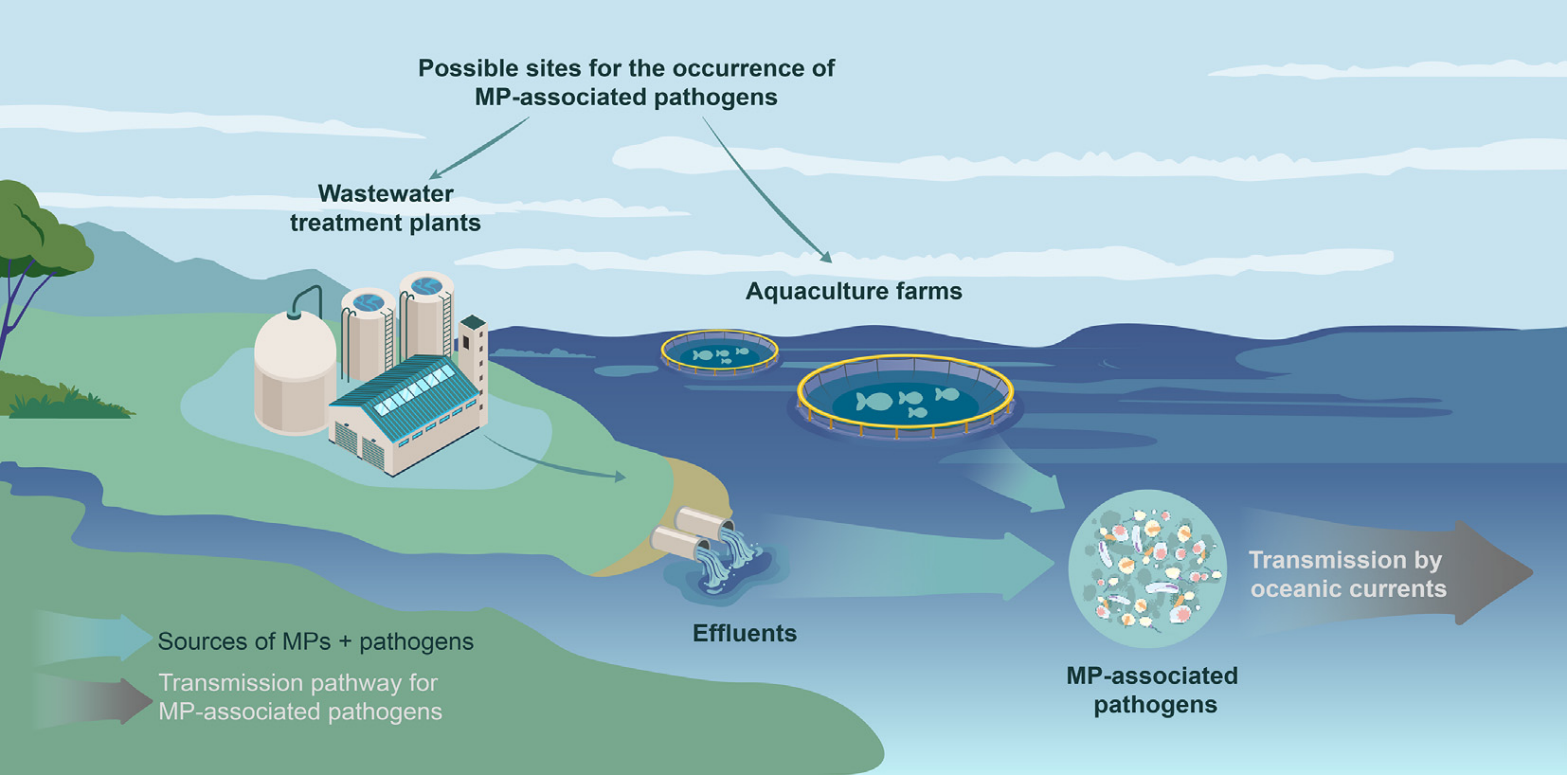
Transmission of MP-associated pathogens in realistic environment.

Transmission of pathogens via MPs is also likely to occur in realistic environments such as WWTPs and aquaculture farms (Fig. 4). WWTPs are recognized as a major source of MP discharge, with daily inputs ranging from millions to billions of particles [89]. Despite disinfection processes, high levels of pathogens, including *Arcobacter* genus, hepatitis E virus, and SARS-CoV-2, can persist in effluents of WWTPs [[90], [91], [92], [93], [94]]. This provides an opportunity for pathogens to associate with MPs, which can then be transported through rivers and oceans, potentially reaching humans.

In Brazilian mussel farming, a study found a concentration of 16.4 MP/m^3^, with a significant presence of synthetic fibers [95]. This is consistent with previous studies by Mathalon and Hill [96], which revealed increased levels of MP fibers in farmed mussels compared to their wild counterparts, suggesting increased ingestion from aquaculture sources. The crowded conditions in aquaculture farms create an ideal environment for pathogen spread, exacerbated by the use of antibiotics [97], which can lead to the development of antibiotic-resistant bacteria. MPs can act as vectors for pathogens by providing attachment surfaces, being ingested by aquatic animals, and facilitating pathogen transport within farms. The presence of MPs in aquaculture facilities poses a risk of disease outbreaks and economic losses [15], highlighting the need for strategies to reduce the presence of MPs and control pathogens.

### 4.2 Factors affecting pathogen transmission

Pathogens, hosts, and the environment are the three main factors that influence successful pathogen transmission to living organisms via MPs. First, pathogen stability and pathogen protein expression and modification are dominant pathogen factors, which determine the persistence of pathogens on MPs and the subsequent transmission with MPs [98]. The stability of pathogens reflected by their half-lives is usually affected by their composition and structure [99]. For example, compared to DNA viruses (e.g., varicella-zoster virus), RNA viruses (e.g., influenza viruses) have a higher mutation rate, which allows them to better adapt to changing environments and thus enhance transmission efficiency [100]. Aside from pathogen stability, the expression and modification of proteins play a vital part in controlling the sites of infection in hosts and the interaction between pathogens and hosts [101]. For instance, the efficient transmission of avian influenza to humans is hypothesized to be associated with the optimal ratio of hemagglutinin to neuraminidase [102]. With respect to the hosts, crowding and co-infection are the primary host factors promoting the transmission in the case of MP-associated pathogen transmission to humans [103]. Increasing the host population may lead to changes in pathogen prevalence and increases in pathogen pressure and the contact rate of humans and MPs [104]. Co-infection induces within-host competition between different pathogens, potentially increasing the rate of pathogen transmission [105,106]. Finally, environmental factors play a dual role in shaping the transmission of MP-associated pathogens. Appropriate environmental factors, such as low temperature, increase the survival and persistence of pathogens after release from MPs [107]. The average half-life of SARS-CoV-2 in surface sputum has been shown to be 5.8 h at 4 °C, significantly longer than that at higher temperatures (3.1 h at 27 °C) [108]. Additionally, environmental factors can modulate host behavior, thus affecting pathogen transmission [101]. Higher temperatures can make the most susceptible species more susceptible [109], while abnormally cool periods can enhance the transmission possibility for certain pathogens, such as the chytrid fungus, to amphibians from warm regions when conditions become cool, compared to those from cooler regions [110].

It is worth noting that the survival time of pathogens also plays a significant role in determining the health risks associated with MP-associated pathogens. However, assessing whether the transport time of MPs exceeds the survival time of viruses on surfaces is a complex endeavor due to multiple influencing factors, including the specific types of pathogens and the prevailing environmental conditions. The precise impact of environmental conditions on the survival of MP-associated pathogens remains relatively limited in our current knowledge. Understanding how factors such as temperature, salinity, pH, and other environmental parameters influence the survival dynamics of pathogens on MP surfaces requires further dedicated research efforts. Addressing this challenge calls for focused studies aimed at unraveling the intricate relationship between environmental conditions and the survival of pathogens on MPs. By conducting comprehensive investigations, we can gain deeper insights into the mechanisms underlying pathogen persistence on MP surfaces and refine our understanding of the potential health risks involved. By shedding light on the effects of environmental conditions, future research endeavors will help us develop a more robust framework for assessing the risks associated with MP-associated pathogens. This will contribute to informed decision-making processes and aid in implementing appropriate measures to mitigate and manage these risks effectively.

## 5 Human exposure pathways and potential infection risk

### 5.1 Possible infection pathways

**Fig. 5:**
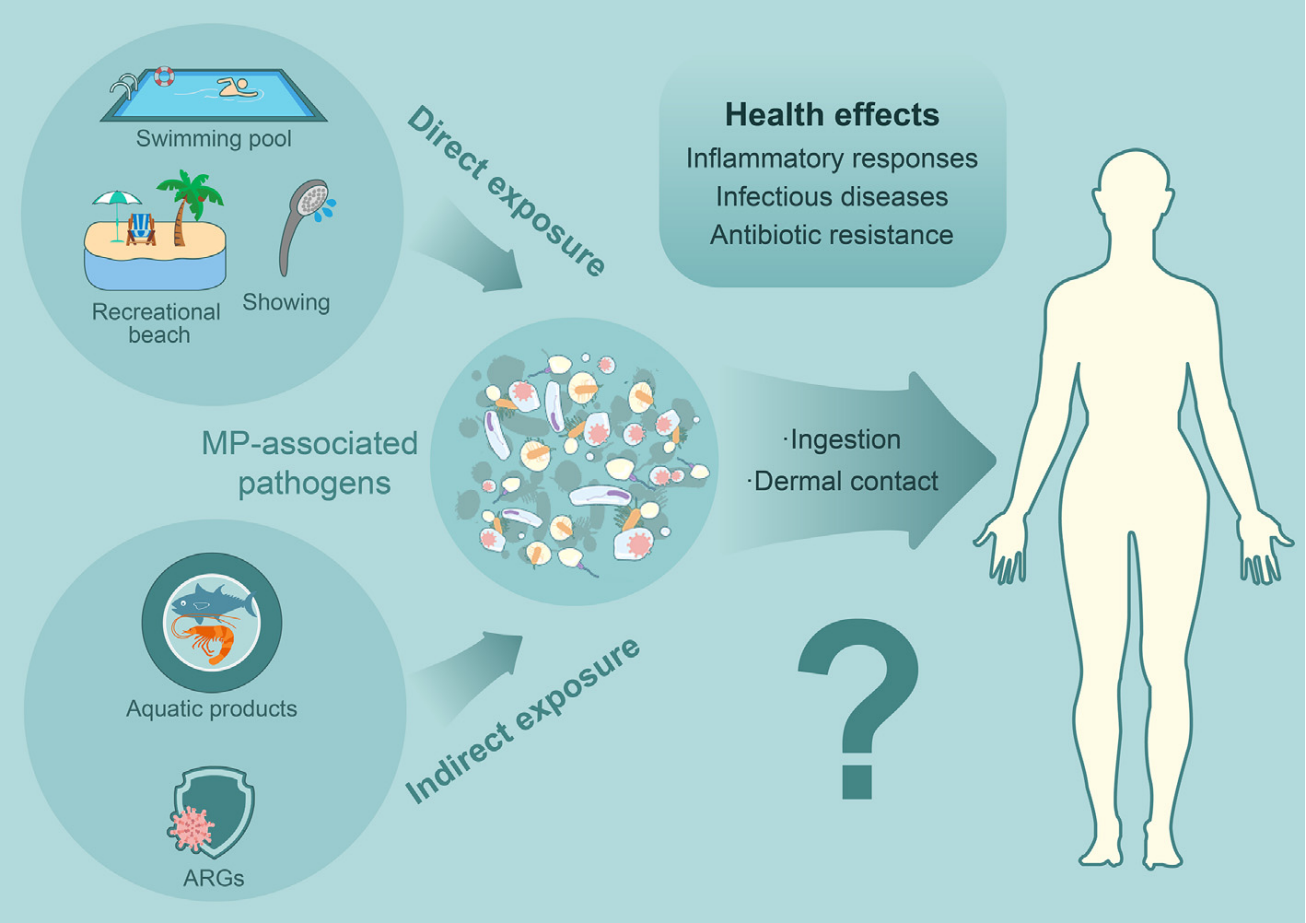
Routes for human exposure to MP-associated pathogens and potential health effects.

It is crucial to comprehend the potential exposure routes of MP-associated pathogens to humans to assess the health risks they may pose. The exposure pathways to MP-associated pathogens can be categorized into direct and indirect routes (Fig. 5). Direct exposure occurs through ingestion of contaminated water either unintentionally consumed or swallowed during activities such as swimming. In addition, direct contact with the skin, particularly when it is damaged, during showering or swimming, can contribute to exposure [111].

Swimming pools and recreational beaches where pathogens and MPs may accumulate are likely hotspots for direct human exposure to MP-associated pathogens. There are two main reasons. First, various pathogens, including *E. coli*, *Cryptosporidium*, *Giardia*, and noroviruses, are present in such settings due to human clustering [112]. For example, the average concentrations of viable *Cryptosporidium* in recreational lakes varied between 0.02 and 0.07 oocysts/L, and the average concentrations of viable *Giardia* ranged from 0.01 to 0.14 cysts/L [113]. *Cryptosporidium*, for example, can survive for more than seven days in well-maintained pools, despite the use of disinfectants [114]. Second, swimming pools and recreational beaches serve as non-negligible sources of MPs because of the frequent use of plastic items, such as polyamide and spandex swimsuits, which may release MPs into the water [115]. While it is widely known that MPs can harbor pathogens, the extent of the risk associated with swimming pools and recreational beaches is poorly understood. Nevertheless, the co-occurrence of MPs and pathogens in swimming pools and recreational beaches necessitates further investigation to better assess the potential health implications.

Indirect human exposure to MP-associated pathogens may also occur, primarily through the food chain. There is increasing evidence that humans are exposed to MPs by ingesting various aquatic products such as fish, shellfish, and seaweed [[116], [117], [118]]. Human ingestion of MPs is further suggested by the recent discovery of MPs in human placenta and feces [119,120]. Aquatic organisms may become infected when exposed to MP-associated pathogens, likely to be transmitted to humans via the food chain. Plastic debris in the marine environment has been found to contain isolates of the fish pathogen *Aeromonas salmonicida*, which can express virulence factors associated with secretion systems and carry ARGs, raising concerns about the potential for MPs to act as disease vectors in aquaculture [121]. Preliminary evidence suggests that the abundance of pathogen-associated plastic debris in the Asia–Pacific region is linked to increased infection rates in corals, indicating that pathogens can be transferred from plastics to aquatic organisms [122]. Laboratory studies have also demonstrated the role of MPs as pathogen vectors. For example, biofilmed MPs colonized with *E. coli* have been shown to infect the temperate coral *Astrangia poculata*, highlighting the potential of MPs to spread pathogens [123]. Furthermore, MPs have been reported to harbor pathogens affecting aquatic organisms such as fish, crustaceans, and mollusks. Larger fish species at higher trophic levels, which are consumed after the removal of their gastrointestinal tract, posing a relatively low risk to humans. Nonetheless, small fish and shellfish, which are generally consumed as a whole, may increase the risk of human infection because MPs do not biomagnify in most cases and are more prevalent in lower trophic organisms [124]. Although human infection caused by MP-associated pathogens via the food chain is possible, aquatic processing, such as cooking, can effectively reduce the risk by wiping out pathogens. However, fisheries and aquaculture workers may be at a hidden risk of exposure to MP-associated pathogens.

In addition to the concerns posed by pathogens, antibiotics on MPs are becoming increasingly worrisome due to their propensity to serve as reservoirs for ARGs from the aquatic environment and their selective accumulation of these genes [125]. Notably, some types of MPs have been shown to exhibit selective concentrations for antibiotic resistance. For instance, polyvinyl chloride particles with the size of 1 μm can adsorb levofloxacin at concentrations up to 1.48 mg/g in an aquatic solution, while polyamide MPs have maximum adsorption capacities for antibiotics, including ciprofloxacin, trimethoprim, tetracycline, and amoxicillin, ranging from 0.1 to 3 mg/g [126,127]. These findings suggest that MPs have the potential to selectively enrich ARGs. Empirical evidence has demonstrated that MPs are hotspots for ARGs, with a significantly higher prevalence of these genes in microbiomes extracted from MPs compared to those isolated from surrounding seawater. Moreover, MPs have been identified as sites that foster greater opportunities for gene exchange. For example, Wang et al. [125] found a significant correlation between the profiles of ARGs and the composition of bacterial communities, indicating that MPs play a selective role in the enrichment of ARGs. Consequently, the co-existence of substantial quantities of antibiotics and bacteria on MP surfaces may facilitate the dissemination and exchange of ARGs [128]. This could exacerbate the proliferation of antibiotic-resistant bacteria and expedite their transmission, ultimately resulting in the transfer of these pathogens with antibiotic resistance up the trophic chain to humans [129]. Despite these concerning findings, the repercussions of oral exposure to MPs contaminated with ARGs, particularly during *in vitro* human digestion models or *in vivo* experiments on mammals, have yet to be comprehensively investigated. As such, further investigation is necessary to fully understand the implications of MP-mediated ARG transmission and to inform mitigation strategies.

### 5.2 Potential adverse impacts

Human ingestion of MP-associated pathogens from aquatic environments can potentially harm human health. These adverse effects result from interactions between the ingested pathogens and the human body, leading to a variety of physiological, immunological, and toxicological responses [130]. While the specific consequences may vary depending on the pathogen species and individual susceptibility, several potential adverse effects can be anticipated.

One potential adverse effect of human ingestion of MP-associated pathogens is the development of infectious diseases. Pathogens such as bacteria and viruses have the potential to colonize and proliferate within the human body, causing infections that may manifest as gastrointestinal illnesses or other systemic infections [131]. The severity of these infections can vary depending on the specific pathogen involved, its virulence factors, and the individual’s immune response. In addition to direct infection, MP-associated pathogens can contribute to the spread of ARGs [40]. Pathogens residing on MPs may carry genetic elements that confer antibiotic resistance, which can be transferred to other microorganisms within the human gut microbiota or to pathogens in the environment. This transfer of ARGs has implications for the effectiveness of antibiotic treatments and the overall management of infectious diseases [40]. Furthermore, the ingestion of MP-associated pathogens can trigger inflammatory responses in the human body [131]. Pathogens can induce immune responses that result in the release of pro-inflammatory cytokines and the activation of immune cells [132]. Prolonged or excessive inflammation can contribute to chronic diseases like gastrointestinal disorders or even systemic diseases like cardiovascular disease.

It is important to note that the extent and severity of these adverse effects may depend on various factors, including the pathogen load, exposure duration, individual susceptibility, and co-occurring risk factors. Further research is needed to better understand the specific mechanisms and effects of MP-associated pathogens on human health, allowing for more accurate risk assessment and the development of appropriate mitigation strategies.

### 5.3 Infection risk

Whether human exposure to MP-associated pathogens leads to subsequent infection in real-world scenarios remains a critical issue requiring clarification. An accurate quantitative assessment of the risks of MP-associated pathogens is currently lacking. However, in a precautionary manner, considering the worst-case scenario, the assessment of potential health effects involves the setting of a safety threshold. The minimal dose required for infection or the observation of adverse effects, as well as the maximum number of pathogens on the surfaces of MPs, are vital factors to determine the likelihood of adverse effects. Due to limited available data regarding absolute concentrations of pathogens on MP surfaces, our analysis primarily focuses on the estimation of health risks associated with *E. coli* as an illustrative example. According to previous research, the minimum infectious dose for *E. coli* has been reported as 10^5^ colony-forming units (CFU) [133]. In the context of MP surfaces, the observed loads of *E. coli* have been found to be approximately 10^4^ CFU per particle in wastewater settings [134]. Considering the typical removal efficiency of pathogens in water treatment processes, estimated to be around 99% [135], the minimum number of *E. coli*-loaded MPs consumed per individual is projected to be 1,000 particles. However, it is crucial to acknowledge that this estimation and safety threshold are subject to uncertainties arising from various factors, including variations in pathogen types, MP characteristics, and individual susceptibility, highlighting the need for further research and quantitative assessment in this area.

It should be noted that the mere attachment and transmission of pathogens to MPs do not necessarily result in infections. Taking viruses as an example, to cause a viral infection via waterborne MPs, pathogenic viruses freshly released from a virus-contaminated environment must adhere to MPs and remain viable. Moreover, a susceptible host would need to ingest a sufficient dose of virus-attached MPs before the viruses on MPs are inactivated to cause an infection. Therefore, further investigation is imperative to determine the potential for MP-associated pathogens to cause human infection through experimental and clinical approaches, such as detecting pathogen loads on MPs and assessing the infection effects on humans.

## 6 Conclusions and future perspectives

The complex interactions among pathogens, vectors, hosts, and environmental factors in aquatic environments, coupled with the lack of direct evidence of the transmission of MP-associated pathogens to humans under complex scenarios, hinder the assessment of the risks of MP-associated pathogens to human health. Addressing these knowledge gaps requires interdisciplinary collaboration involving fields such as ecotoxicology, microbiology, and epidemiology. This includes determining the infectious pathogen load on MPs and overcoming technical challenges in pathogen sampling and detection. Investigating pathogen ecology and understanding interactions between pathogens, the environment, and human hosts can provide insights into pathogen dynamics and control strategies. Additionally, establishing accurate mathematical models is essential for predicting disease outbreaks and understanding infection dynamics in different scenarios.

Studying interactions among MPs, pathogens, and humans provides valuable insights into policymakers in public health and environmental management. Reducing MP pollution, particularly in MP and pathogen-rich environments, such as WWTPs and aquaculture farms, is a fundamental strategy. Broader commitments from environmental policymakers are needed to facilitate higher recycling rates of plastic waste and ultimately scale down the discharge of plastics into the environment.

## Author contributions

M.J.W. and H.Z. identified the topic of this paper. M.J.W. collected and analyzed data. H.Z. and H.Q.R. supervised this work. M.J.W. and Y.L.J. interpreted the results and designed the figures. M.J.W. wrote the original draft. H.Z., C.S., S.S.L., R.W.M.K., H.H.S., R.J., X.L.Z., X.M.S., X.X.Z., C.J.L., Y.Y.L., G.B.Q., and F.J. edited the manuscript. All authors discussed the results and reviewed the manuscript.

## Declaration of competing interest

The authors declare no competing interests.
